# A proposed correction in the weighted method to score the Ishihara test

**DOI:** 10.1186/s13104-019-4320-2

**Published:** 2019-05-28

**Authors:** Leticia Miquilini, Mauro Augusto Souza Ratis, Monica Gomes Lima, Natali Valim Oliver Bento-Torres, Eliza Maria da Costa Brito Lacerda, Maria Izabel Tentes Cortes, Anderson Raiol Rodrigues, Luiz Carlos de Lima Silveira, Givago da Silva Souza

**Affiliations:** 1 Universidade Federal do Pará, Instituto de Ciências Biológicas, Belém, Pará Brazil; 2 Universidade do Estado do Pará, Marabá, Pará Brazil; 3 Universidade Federal do Pará, Instituto de Ciências da Saúde, Belém, Pará Brazil; 4 Universidade Ceuma, São Luís, Maranhão Brazil; 5 Universidade Federal do Amapá, Macapá, Amapá Brazil; 6 Universidade Federal do Pará, Núcleo de Medicina Tropical, Av. Generalíssimo Deodoro 92, Umarizal, Belém, Pará 66055240 Brazil

**Keywords:** Ishihara test, Visual psychophysics, Colour vision, Visual perception, Diagnostic test

## Abstract

**Objective:**

Ishihara test is a color vision test, whose results consider that all plates of the test have the same weighting. Rodriguez-Carmona et al. (Aviat Space Environ Med 83:19–29, 2012) proposed an equation to quantify the Ishihara test results (severity index), which took an account the rate of hits from the different plates of the test considering the performance of trichromat or colorblind population. We proposed a correction in Rodiguez-Carmona’s equation for the severity index. We evaluated 60 normal trichromats and 107 subjects with congenital color deficiency. We calculated three indexes to quantify the results of each subject: a non-weighted index, a weighted index similar to the Rodriguez-Carmona et al., and a weighted index modified which combined the hit frequency for each plate in a trichromat population and of the error reading frequency for each plate in color-blind populations.

**Results:**

Compared to the non-weighted evaluation, the weighted index was reduced by 22.95%, 32.92%, and 35.38% from trichromats, protan and deutan groups, respectively. Receiver Operating Characteristics (ROC) analysis showed perfect performance of the classifier for all metrics to measure the Ishihara test results. The proposal correction changed significantly the value of the index, but the overall benefits were small.

**Electronic supplementary material:**

The online version of this article (10.1186/s13104-019-4320-2) contains supplementary material, which is available to authorized users.

## Introduction

The Ishihara test is widely used to evaluate human color vision [1–7]. The test is composed by pseudoisochromatic plates and the perceptual task is dependent of the chromatic differences between the stimulus target and background [7]. Some agencies consider a different number of errors to label the subject as congenital colorblind [4], and the accuracy of the Ishihara test has been the focus of many investigations [1, 2, 4, 8–15] since different plates have different hit rates for trichromat subjects [1, 2, 4, 8–14].

Although the probability of correct response to each plate is different, the current evaluation of the Ishihara test considers that all the plates have the same weight, and the sum of hits for the task is the result of the test. Rodriguez-Carmona et al. [4] introduced an evaluation of the Ishihara test based on the probability of error from each plate. They called it severity index. For a normal trichromat, it considered the probability of correct response to a plate in a normal trichromat population to weight its contribution to the final result. They applied the same rationale to protan and deutan (congenital colorblind) subjects.

Considering that a perfect plate to separate normal trichromat and other color vision phenotypes would be the that which the normal trichromat makes the correct decision and the subject with a color vision deficiency makes the wrong decision, it is reasonable that both pieces of information should be included in the equation of the severity index. In the present study, we introduced a correction in the equation of Rodriguez-Carmona et al. [4] to quantify the severity index of the Ishihara test result.

## Main text

### Methods

#### Subjects

Sixty normal trichromats and 107 congenital dichromats, consisting of 42 protans and 65 deutans, made up our database. The mean age of normal trichromat subjects was 23.78 ± 7.24 years-old, and that of dichromat subjects was 32.72 ± 10.84 years-old. We evaluated both eyes, but we randomly chose one eye to analyze the results. All subjects had normal or corrected visual acuity higher than 20/30 without neurological or systemic diseases. No subjects’ corrective lense had any tint.

#### Ishihara test application

**Fig. 1:**
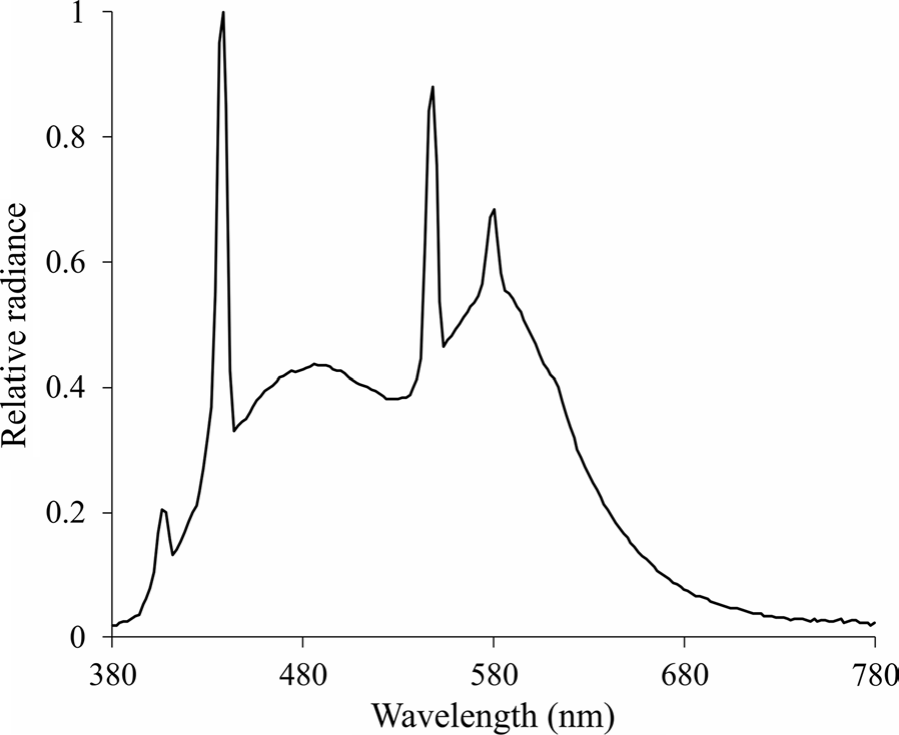
Relative spectral radiance of the white fluorescent bulb used to illuminate the Ishihara plates during the test

We used 38 plates for the Ishihara test (1997 edition) [16]. We used the plates from 1 to 25 to test the subjects. The plates were 75 cm apart from the subjects’ eyes under the illumination of fluorescent lamps, which had relative spectral radiance showed in the Fig. 1. We used spectroradiometer (PR715 model, Photo Research, NY, USA) driven by SpectraWin 2 to record the spectral radiance of the light. We positioned the book around 45º from the table, and the exhibition of each plate lasted 3 s. The subject was instructed to read the number on the plate during its presentation. We compared the results for each plate to the responses indicated by the book manufacturer, and a lack of reading or misreadings were considered errors. We analysed the plates from 2 to 21 to estimate the test accuracy and to apply mathematical analyses, and plates from 22 to 25 to classify the color-vision phenotype of the participants. To be considered colorblind, the subject had to make 8 or more errors on the Ishihara test, and in order to classify subjects as deuteranopic and protanopic, we used the classification plates (Plates 21 to 25) of the Ishihara test. The classification indicated by the Ishihara test was confirmed by at least one more color vision test (Farnsworth-Munsell 100 hue test or HMC anomaloscope).

#### Data analysis: indexes calculation

We analyzed the results of Ishihara test for each subject by calculating a non-weighted index and two weighted indexes. We attributed the value 1 to the plates with correct responses while to the plates with the wrong responses, we attributed the value 0. The non-weighted index was calculated by the sum of the attributed values of the subjects’ responses to the conventional evaluation of the Ishihara test. The value of the sum was divided by the number of plates (Eq. 1).

1 $$Non{-}weighted\;index = \frac{{\sum\nolimits_{i = 2}^{21} {R_{i} } }}{n}$$

where $R$ is the response for each plate (1 for correct, 0 for incorrect response), $i$ is the number of the plate, $n$ is the total number of plates.

The weighted indexes were calculated as follows:

i. Using the equations suggested by Rodriguez-Carmona et al. [4]. For each plate, we also attributed the value 1 for a correct response and value 0 for the wrong responses. After, if the subject was a normal trichromat, we multiplied the value of the subject’s response to the hit rate of the plate in a normal trichromat population. If the subject was protan or deutan, we multiplied the value of the subject’s response to the hit rate of the plate in a population with the same color vision phenotype. We summed up the weighted response to all plates, and the total value were divided by the total number of plates (Eqs. 2a, 2b, 2c).
  2a $$Weighted\;\;index\_i_{t} = \frac{{\mathop \sum \nolimits_{i = 2}^{21} R_{i} \times PT_{hit} }}{n}$$
  2b $$Weighted\;\;index\_i_{p} = \frac{{\mathop \sum \nolimits_{i = 2}^{21} R_{i} \times PP_{hit} }}{n}$$
  2c $$Weighted\;\;index\_i_{d} = \frac{{\mathop \sum \nolimits_{i = 2}^{21} R_{i} \times PD_{hit} }}{n}$$
  where $Weighted\;\;index\_i_{t}$ is the weighted index for normal trichromats based in Rodriguez-Carmona et al. [4], $R$ is the response for each plate (1 for correct, 0 for incorrect response), $i$ is the number of the plate, $n$ is the total number of plates, $PT_{hit}$ is the probability of correct response in a trichromat population, $Weighted\;\;index\_i_{p}$ is the weighted index for protan subjects based in Rodriguez-Carmona et al. [4], $PP_{hit}$ is the probability of correct response in a protan population, $Weighted\;\;index\_i_{p}$ is the weighted index for deutan subjetcs based in Rodriguez-Carmona et al. [4], $PD_{hit}$ is the probability of correct response in a protan population.
ii. Adjusting the equations suggested by Rodriguez-Carmona et al. [4] including the information of normal trichromat and dichromat populations. For each plate, we also attributed the value 1 for a correct response and value 0 for wrong responses. After this, we multiplied the value of the subject’s response to the hit rate of the plate in a normal trichromat population and to the error rate of the plate in a protan and deutan population. We summed up the weighted response to all plates, and the total value was divided by the total number of plates (Eq. 3).
  3 $$Weighted\;\;index = \frac{{\mathop \sum \nolimits_{i = 2}^{21} R_{i} \times PT_{hit} \times PP_{error} \times PD_{error} }}{n}$$
  where $R$ is the response for each plate (1 for correct, 0 for incorrect response), $i$ is the number of the plate, $n$ is the total number of plates, $PT_{hit}$ is the probability of correct response in a trichromat population, $PP_{error}$ is the probability of wrong response in a protan population, and $PD_{error}$ is the probability of wrong response in a deutan population.

We applied the descriptive statistics to the scores of non-weighted and weighted indexes using the results of all Ishihara test plates and using the more efficient Ishihara test plates to each group. The significance level was 5%. Statistical analysis was performed using the software Biostat 5.0.

### Results

#### Accuracy of the test: hit rate

Table 1 shows the hit rates of the Ishihara plates. The normal trichromat subjects obtained 100% of the correct response to the plates 2, 3, 4, 7, 8, 11, and 16. The plates with lowest hit rates were 12, 17, 19, 20, 21, ranging between 66.7 and 75%. There were 26.67% of normal trichromat subjects that showed 100% hits on all plates.

**Table 1:** Hit rate to each plate of Ishihara test

Hit rate (%)				
Design	Plate	Normal trichromat	Protan	Deutan
Confusion design	2	100	7.1	1.5
	3	100	11.9	6.2
	4	100	2.4	1.5
	5	96.7	0	0
	6	98.3	0	9.2
	7	100	9.5	35.4
	8	100	2.4	21.5
	9	96.7	0	1.5
Vanishing design	10	98.3	2.4	0
	11	100	0	0
	12	73.3	0	0
	13	88.3	0	0
	14	98.3	0	0
	15	98.3	2.4	1.5
	16	100	0	0
	17	75	0	0
Hidden digit design	18	88.3	9.5	23.1
	19	73.3	9.5	10.8
	20	66.7	9.5	12.3
	21	73.3	14.3	20

For the protan group, we observed no (0%) hit on plates 5, 6, 9, 11, 12, 13, 14, 16, and 17 and the plates 3 and 21 had higher hit rates. For the deutan group, the plates that showed hits of 0% were 5, 10, 11, 12, 13, 14, 16, and 17, and the plates with the highest hit rates for deutans were 7, 8, 18, 19, 20, and 21.

#### Comparison of the non-weighted and weighted indexes

**Fig. 2:**
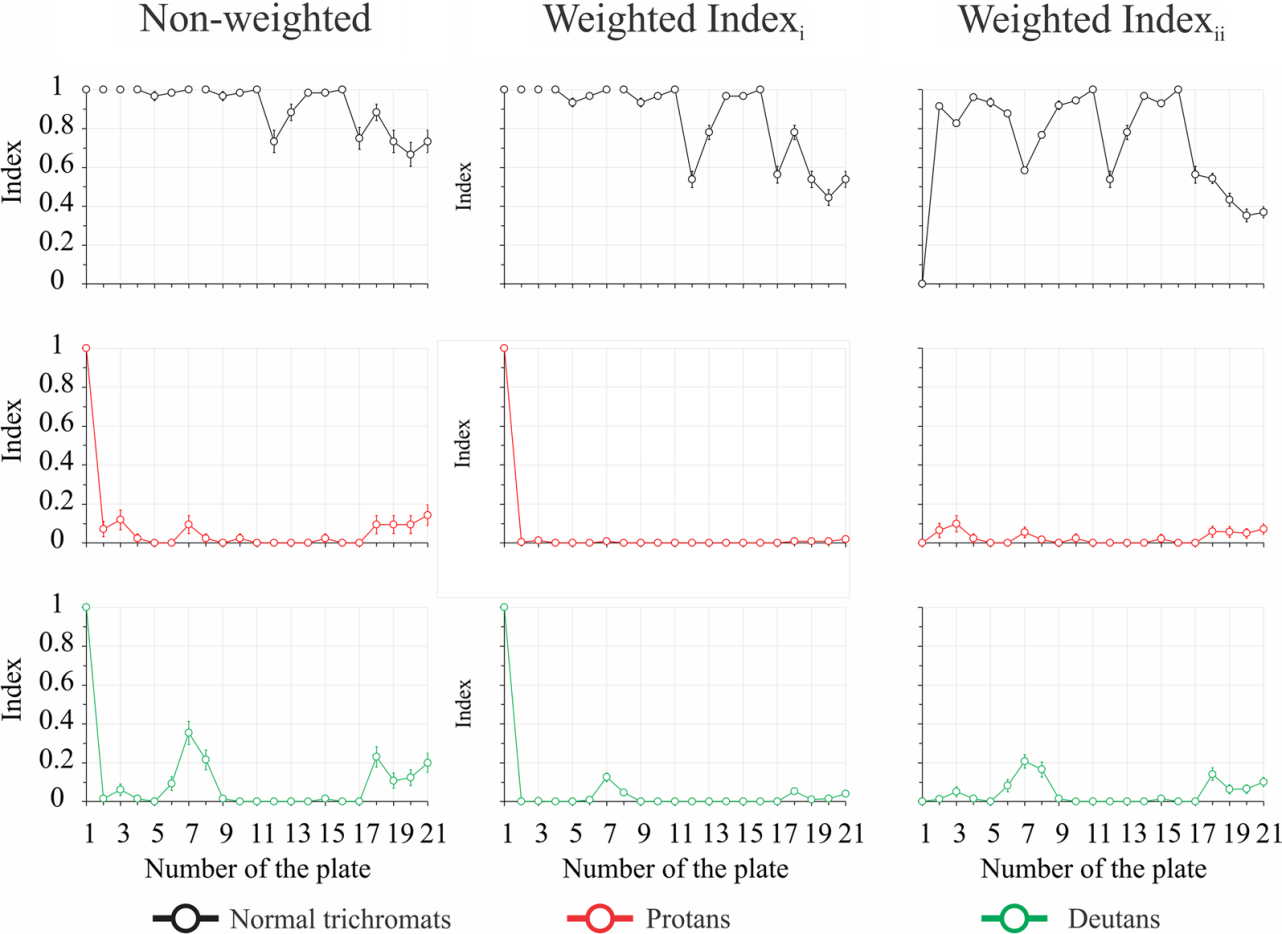
Indexes estimated from normal trichromats (black circles and lines), protan (red circles and lines), and deutan (green circles and lines) populations for each plate using non-weighted method (conventional evaluation), weighted_i_ method (based in the Rodriguez-Carmona’s equation), weighted_ii_ method (modified from the Rodriguez-Carmona’s equation)

Figure 2 shows the partial index for each plate calculated by non-weighted and weighted equations. Comparing the functions obtained for each index, we observed that the values of the indexes in some plates showed noteworthy differences.

The normal trichromat group exhibited higher index values than the groups with congenital color vision deficiency (p < 0.05) while there were no differences between the groups with congenital colorblindness (p > 0.05). The weighted index values were significantly lower than the non-weighted values for all groups (p < 0.05).

#### Receiver Operating Characteristic (ROC) analysis

We performed the ROC analysis to evaluate the performance of the classifier that used the different ways to measure the severity indexes used in the present investigation. For the ROC analysis, we considered the cumulative distribution of the indexes estimated from the trichromats as true positive rates, and we considered the indexes estimated from each group of subjects with color vision deficits as the false positive rates. The area under curve was calculated using MATLAB routines with the trapezoid function, and we observed that for all indexes the area under curve was 1.

### Discussion

The present investigation proposed to modify the Rodriguez-Carmona’s equation [4] to calculate the severity index of the Ishihara test results. In our modification, we included information of normal trichromats and congenital colorblinds. Our main results were that for some plates, the proposal correction changed significantly the value of the index (for example, plate 1), but the overall benefits were small. We interpreted that the data distribution between congenital colorblind subjects and normal trichromats are largely separated, and all the methods to calculate the severity index we are studying in the present investigation had the same performance (ROC analysis) to distinguish both color vision phenotypes. However, we considered that the rationale of the Rodriguez-Carmona’s equation is partially correct and with our modification would be adequate to be applied for other situations where the distance of the distribution between normal trichromats and subjects with acquired color vision deficiency was smaller.

Many studies have shown that the plates in the Ishihara test lacked the same efficiency to elicit correct responses from normal trichromats and colorblinds [1, 2, 4, 8–15]. Probable reasons for confusing the reading of Ishihara plates are lens pigmentation due to aging [17], abnormal contrast sensitivity [18], decreased visual acuity [19], illuminant [7], and the incorrect illumination and administration of the test [20]. Moreover, variations in color printing between one copy of the test and another may also affect the subject’s ability to read [4, 9, 21, 22].

The non-weighted method is equivalent to the method of quantification suggested by the Ishihara test manufacturer. Rodriguez-Carmona et al. [4] proposed a severity index for the Ishihara test. The advantage of this index was the idea to weight the number of errors in the Ishihara test by the probability of correct responses in particular color vision phenotype populations. The results of a normal trichromat were weighted by the probability of hits on the plate in a normal trichromat population, and protan and deutan subject results were weighted by the probability of hits on their population. The method proposed in the present investigation used the idea of a weighted evaluation of the Ishihara test from Rodriguez-Carmona et al. [4] that we modified to adjust to the idea of what is expected for a perfect plate. We considered that a “perfect” plate would have to be seen by all normal trichromats and not be seen by all subjects with congenital colorblindness. Then, we weighted the responses by the hit probability on normal trichromats and by the error probability of a subject with congenital colorblindness. The implementation of the modification enabled to apply the same equation for any subject (normal trichromat or not), and that was not possible to do using the Rodriguez-Carmona’s equation.

## Limitations

We had a smaller sample size than previous studies [4, 13], but we considered that the increase of the sample size would improve the good separation of the color vision phenotype groups we already observed. Six out of 9 authors were examiners for the color testing, and it could introduce some examiner bias way to apply the test. As all the examiners had the same training, we expect that this bias would not have had a great impact in the results. The fluorescent illumination is not recommended by the manufacturer, but some investigations have discussed about the use of this kind of illumination during the use of Ishihara test and they have indicated some influences on the results [11, 23]. Ishihara test was designed for natural daylight or CIE standard illuminant C and International standards for colour vision testing have been given [24]. Fluorescent bulbs have pronounced energy in some wavelengths and differ from natural daylight that has similar energy distribution across spectrum [25]. The comparisons between the results of pseudoisochromatic test using natural daylight (or Macbeth easel lamb) and fluorescent daylight lamp have shown variable results [11, 25, 26]. For the present study, some influence of the fluorescent lamp was minimized by the confirmation of the color vision phenotype by other color vision tests.

All the limitations of the present study had little or none impact in the differences between our results and those from Rodriguez-Carmona et al. [4], since that the accuracy of the results for trichromats and dichromats is similar to those showed in previous study [1]. We consider that the differences between both studies was due the different ways to calculate the weighted methods to quantify the Ishihara test performance (Additional file 1).

## Additional file

**Additional file 1.** Binary results of Ishihara test. Three spreadsheets containing the binary results of Ishihara plates for normal trichromats (spreadsheet #1), protan subjects (spreadsheet #2), and deutan (spreadsheet #1). Value 1 means correct response, value 0 means incorrect response.

## Data Availability

The datasets the current study is available from the corresponding author on request by the e-mail: givagosouza@ufpa.br.

## Abbreviations

ROC: Receiver Operating Characteristics
SIN: screening inefficiency number
NT: normal trichromats
P: protan
D: deutan
Q1: first quartile
Q3: third quartile
P2.5: percentile 2.5%
P97.5: percentile 97.5%
